# Identifying the Service Capability of Long-Term Care Facilities in China: An e-Delphi Study

**DOI:** 10.3389/fpubh.2022.884514

**Published:** 2022-06-29

**Authors:** Wen Liu, Min Hu, Wen Chen

**Affiliations:** Health Economics Department, School of Public Health, Fudan University, Shanghai, China

**Keywords:** long-term care facilities, long-term care insurance pilot, service capability, Delphi consultation, index system

## Abstract

**Objective:**

This study develops a group of service capability indicators for long-term care facilities to assess their current conditions and makes it the first step toward the improvement of service capability in China.

**Methods:**

We constructed an initial indicator framework based on the characteristics of long-term care services and a literature review. Potential indicators were collected, and a 2-round modified web-based Delphi process was conducted by a national multidisciplinary expert panel to construct a service capability evaluation index system. The accepted competencies of indicators were established with mean scores in all three scoring criteria (importance, feasibility, and sensitivity) ≥ 4.0, consensus rate reached 70.0%, and a coefficient of variation ≤ 0.25.

**Results:**

A new indicator framework covering 2 dimensions of inputs and activities was developed in this study. The initial 35 indicators formed an indicator pool for the Delphi questionnaire. According to the final consensus of the expert panel, the Delphi consultation resulted in an index system comprised 31 tertiary indicators across six subdimensions (i) staffing; (ii) facilities and equipment; (iii) funding; (iv) medical inspection services; (v) health management services; (vi) institutional standard management.

**Conclusion:**

This study developed a set of indicators suitable for the long-term care system in China and is expected to be applied to measure and improve the service capability of long-term care facilities. In addition, these indicators can be used for comparisons between different LTCFs and provide an evidence basis for the further development of capability assessment tools.

## Introduction

Older adults aged 60 and over accounted for 18.70% of the Chinese population in 2020 (1), and 42 million (15.91%) of them experienced physical limitations. In 2020, the disability rate of the population aged 85 and above reached 34.7%, and the prevalence of chronic diseases displayed the same growth trend (2, 3). It is expected that the physical restrictions that older adults face as a result of progressive diseases and functional deficits will continue to escalate with the aging and increased longevity of the population. Consequently, the need for long-term care (LTC) services among those people are expected to increase dramatically (4). In other words, service improvement is important and indispensable for older people, which means a well-designed LTC system and effective management along with quality assurance (5, 6). LTC services refer to a variety of services that help meet both the medical and non-medical needs of people who cannot care for themselves over a long period (usually 6 months). In particular, it provides help in activities of daily living (ADLs), such as bathing, dressing, toileting, and walking, and in instrumental activities of daily living (IADLs), such as housekeeping, shopping, preparing meals, and managing money (7). These services may be provided in either institutional settings such as long-term care facilities (LTCFs) or in non-institutional settings such as older adults' homes or communities. Services received from paid caregivers are termed “formal care” (8, 9). Generally, LTCFs provide specialized formal care for older people with higher-level care dependence. This represents an important component of overall senior health or welfare policies and has a significant impact on social development (10, 11).

To meet people's growing demand for multilevel LTC services, China launched a systemic pilot program covering 15 cities (called the long-term care insurance pilot, LTCI pilot) in 2016 that took actions such as carrying out disability grade assessment, developing LTC service packages, and reforming LTC payments (12). The second round of this pilot was launched in 2020, which has covered all provinces in China (each province contains at least one pilot city). The existing pattern of LTC services in China includes community home care and institutional care, among which institutional LTC is aimed at elderly individuals with a higher degree of disability (13). Since the implementation of the pilot program, only the seniors with a disability level 4 and above can apply for services from an LTCI-designated institution. A series of guidelines imposed more stringent requirements for service improvement within LTCFs and led them into intensifying competition. LTCFs have thus been exploring their options concerning the questions of how they can maintain their competitive advantage in the LTC market and how they can develop sustainably.

As a professional service for improving health, LTC services have certain similarities with health services, and extensive research has been carried out in the field of health. In recent years, health service research has not only focused on service quality but also gradually explored the issues of service capabilities. The viewpoint of organizational capability theory is that organizations need to focus on continuous capability building to achieve goals and form long-term competitiveness under the influence of the external environment. In other words, the theory suggests that internal factors (capability) are the leading factors in organizational growth and determine its degree and scope. The basic assumption of organizational capability theory is: in a specific external environment, the improvement of organizational capability will be conducive to the realization of its ultimate goal. As an abstract concept, the mechanism of service capability can be explained as follows: capability does not directly present competitive advantages but embodies the process of resource acquisition, allocation, and utilization through a series of structural or procedural elements, that is, the transformation from input, activity (or process) to output (or result) (14). At present, the research on the capability of health services mainly focuses on the perspective of service personnel, that is, personnel competence. The specific literature includes the core competence of health professionals for specific diseases (15), the ability of medical staff to obtain evidence (16–18), information technology capabilities in hospitals (19), and the subjective competence of nurses (such as empathy capabilities in nursing services) (20). Regardless of the dimension of health service capability research, existing studies generally show that the suitability of service capacity has a key impact on health service quality (or subjective and objective outcomes). The research ideas of health service can provide a reference for the in-depth study of LTC service, but the differences between LTCFs and medical institutions still need to be considered: (1) In addition to focusing on the professionalism of formal LTC services, it is also necessary to consider the nursing environment; (2) Nurses in LTCFs may also play the role of service managers; (3) The elderly lives in LTCFs for a long time, even until death; (4) Demanders have higher requirements for maintaining autonomy and body functions^1^.

Based on the research experience in health services, it is necessary to discuss the front-end factors of service outcomes to promote service quality. Therefore, research on LTC service capabilities also requires further attention. The concepts of capability or capability development are so all-encompassing that practitioners have often found it difficult to make operational sense of them. It is important for researchers to begin by asking the question “capability for what?” and focus on the specific capabilities needed to accomplish clearly defined goals. From a general point of view, capabilities describe the functional building blocks that enable service delivery (the “what”). A complete assessment of the capability landscape will take resources, process or other dimensions into consideration. Processes describe how services are implemented. The United Nations Development Programme (UNDP) puts forward that capacity is the ability of environment, organization or individual to achieve corresponding functions, or the power to perform specific functions (21). WHO proposes in LTCFs statistical indicators that facility availability and capacity indicate the ability to provide care to people with dementia and to meet their needs and preferences (22). There is no official or recognized definition of service capability in LTCFs, but existing research on service quality and its influencing factors can provide inspiration for clarifying this topic. A large body of tools are available to evaluate the service quality of LTCFs, including outcome measurement indicators developed by different research teams (23, 24) or nationally unified scale tools (25, 26). Some studies define service quality as a more comprehensive concept, covering environment, resource allocation and other factors (27–29). There is sufficient evidence that staffing (30–34), service process (35, 36), rehabilitation care (37), internal management (38), or training of managers (39) in LTCFs are closely related to service quality. It can be found that although many studies have explored the front-end factors of service quality, they are still discussions on a single dimension. At the same time, the existing literature on LTCF's capability is mostly limited to specific topics, such as the competency of LTC personnel^2^. The WHO has developed a competency framework for rehabilitation personnel (40). There is a lack of comprehensive combined and integrated analyses that would allow effective exploration of the heterogeneity of LTCF's service capability.

Therefore, the current study aims to establish a theory-based indicator framework of service capability for LTCFs and systematically develop a set of indicators specifically suitable for China using an interprofessional Delphi process. The paper's intended audience is a broad range of policy makers and practitioners. But it is directed most particularly to those working on continuous improvement of LTC services at the field level. The resulting set of indicators is intended to be applicable tools for measuring the comprehensive service capability of LTCFs, furthermore, to serve as a framework to guide and stimulate ongoing discussions on service improvement in aging societies and LTC settings.

## Methods

### Generation of New Indicator Framework and Potential Indicators

**Figure 1:**
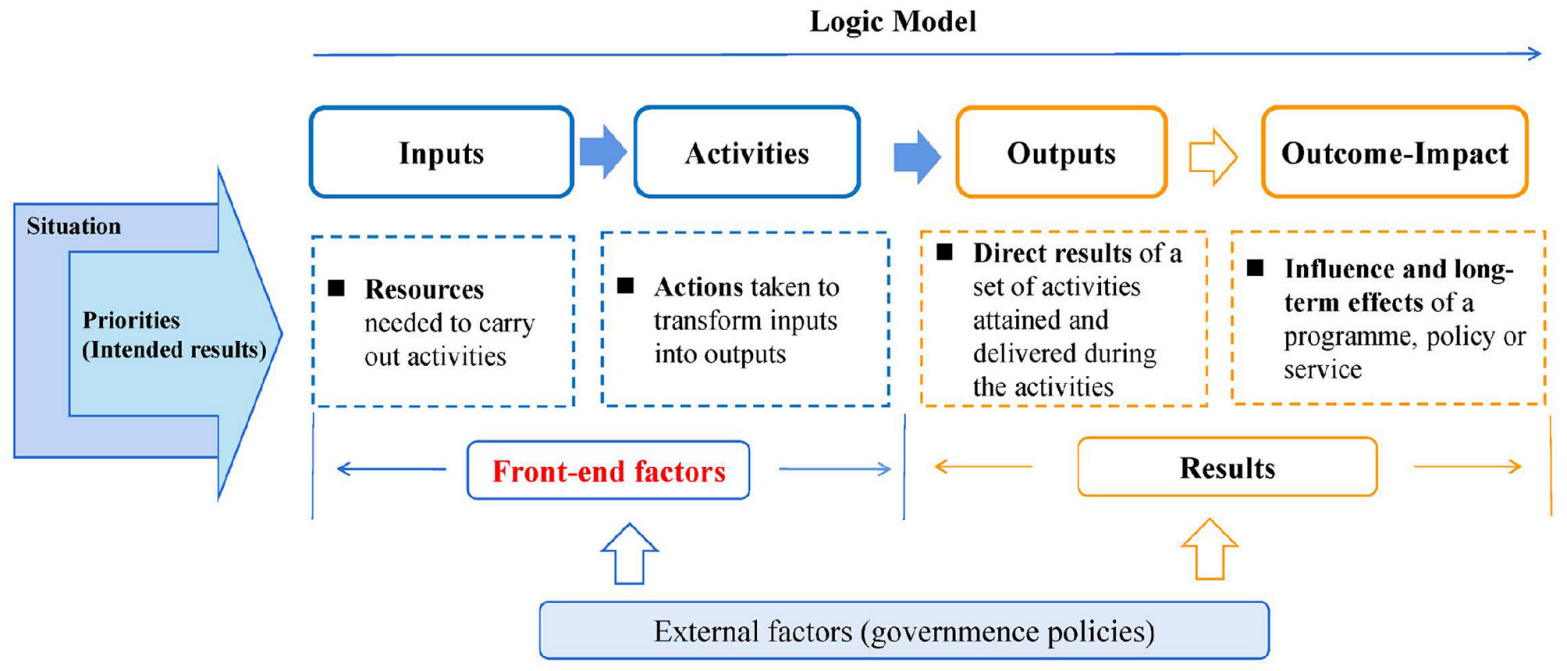
Elements of a results chain.

The classical results chain framework, also known as “logic models,” is often used in indicator development studies. They are diagrams that map out a series of statements that link factors in an “if…then” fashion—for example, if a certain type of service is provided, then a performance of the service organization could be enhanced or if a policy opportunity is taken, then a thematic target might be improved (41). Combined with the existing literature, the inputs are used to carry out activities, these two elements are front-end factors that lead to results, including the services or products delivered (outputs), the immediate change (outcomes), and eventually the long-term impact (42). It is worth noting that this chain should be analyzed in terms of the situation (such as market conditions, policies) and intended results (Figure 1). Combining the aforementioned organizational capacity theory and evidence from the literature on quality of service, in the service organization, the front-end factors could be refined into the concept of comprehensive service capability. For the LTCFs, input describes the resource characteristics of service providers, such as the qualification of the nursing staff or the allocation of related equipment. Activities cover the procedures or methods for medical technicians and nursing staff to provide services, which will be reflected in the quality of care (QoC) if properly used (43). We consider that, similar to professional organizations, in addition to service delivery in LTCFs, their internal management of LTCFs also plays an important role in obtaining better service results. The service capability of an LTCF in our study was defined as follows: by adapting to the external environment (policy and LTC market), the LTCF organizes internal resources to manage daily services and administrative work to effectively operate and provide LTC services (44). Based on this, a new indicator framework including inputs and activities is constructed to guide the development of service capability indicators in LTCFs.

Under the guidance of the indicator framework, national practice guidelines were reviewed to extract recommendations in LTC service improvement as candidate indicators, that is, the initial Delphi instrument. A systematic literature search was conducted in electronic databases using the search terms “long-term care,” “service capability,” “quality of care,” and “performance measure,” which can help us understand the relevant indicators in other countries. In addition, the added items were identified from an expert panel. Their Chinese versions with detailed definitions were prepared to be discussed in the first round.

### Delphi Process

This study used the Delphi method to build a service capability evaluation index system for LTCFs. The e-Delphi technique is an environmentally friendly approach to research that leads to rapid feedback and responses from an expert panel (45). In general terms, this method assumes that the opinion of experts can have a scientific application (46). It consists of a participatory methodology that aims to generate consensus, where the participants building consensus on the subject in question, but without direct confrontation of opinions. To this end, it implies a structured process and a systematic, effective, reliable and comprehensive technique for collecting and distilling knowledge from a group of qualified specialists carefully selected by means of a series of anonymous questionnaires interspersed with controlled feedback. The obtained results, to a large extent, have a multidisciplinary vision and the potential to obtain viable data that allow informing policy makers or other practitioners.

The basic criteria for the selection of experts in our study include (i) expert authority, which means the academic background related to LTC; (ii) a wide range of sources, including scientific researchers and management personnel in LTC-related administrative departments; and (iii) expert qualification, which refers to the professors (or associate professors) with experiences in professional work for more than 8 years.

#### Round 1- Rating of Indicators

The indicators confirmed in the initial part were formulated into a Delphi questionnaire with a letter introducing the background and the aim of the study as well as detailed instructions of scoring criteria for indicators: importance, feasibility, and sensitivity. The rating scale of each indicator was a five-point Likert scale (Table 1). Experts in this study came from a wide range of background expertise and perspectives across different types of organizations. Our interdisciplinary working group are very familiar with domestic LTC-related researches, and has established a database of experts with rich research achievements (mainly from universities/colleges and academic research institutions). Combined with the above principles, 15 candidates in the expert database who met the inclusion criteria of this study were further selected. Potential candidates from pilot cities were recommended by the directors of the Healthcare Security Administration, and five administrative experts were finally selected to participate in the Delphi process. They all had leading positions in their relevant institutions which contributed to the identification of the priority issues during the Delphi process. The questionnaire was distributed by e-mail to the 20 expert panel members, followed by a reminder e-mail 2 weeks later. Modifications, eliminations and combinations were made based on the above considerations, and experts were encouraged to propose new items to existing ones if deemed necessary.

**Table 1:** Example of the Delphi questionnaire.

	**Indicators**	**Importance**	**Feasibility**	**Sensitivity**	**Suggestions**
Dimension 1	Indicator 1				
	Indicator 2				
	Indicator 3				
	Other indicators that need to be added in this dimension (Please explain the reason in detail):____________________				
Dimension 2	Indicator 1				
	Indicator 2				
	Indicator 3				
	Other indicators that need to be added in this dimension (Please explain the reason in detail):____________________				

*(1) Experts need to rate each indicator in the questionnaire on a 1–5 scale; for example, 1 means the least important, and 5 means the most important. (2) Feasibility represents the availability of the corresponding data for this indicator; 1 means that the data are difficult to obtain, and 5 means that the data are easy to obtain. (3) Sensitivity refers to the extent to which the indicator can influence changes in service capability results. The higher the degree of discrimination, the higher the sensitivity score of the indicator*.

#### Round 2- Revision and Grading Based on the First Round of Feedback

After the first survey round, SPSS V.23.0 was used to compute the indicator score. The rating result of each indicator was discussed after the round one feedback. In addition, whether the indicator was suitable for the measurement of the service capability of LTCFs in the environment of the LTC system in China was also discussed. The eliminated indicators in Round 1 were reviewed again to decide whether some of them were also important and could be retrieved. The new questionnaire with revised indicators and detailed scores was sent to the expert in round 1 again. Feedback was received 2 weeks later.

### Data Analysis

The scientific soundness and rationality of the Delphi method are reflected by three indicators: experts' positive coefficient, authority coefficient, and coordination coefficient.

(1) The experts' positive coefficient reflects the effective response rate to the consultation questionnaire and determines the credibility and scientific basis of the results. Authoritative data show that an effective response rate of 50% is the minimum acceptable value for the Delphi method, 60% is considered moderate, and over 70% meets a very good standard (47).
(2) The expert authority coefficient (Cr) is generally determined by two factors: the judgment coefficient (Ca), which represents the evidence for the expert to make a judgment, and the familiarity coefficient (Cs), which represents the expert's familiarity with the issue (48). As shown in Table 2, Ca is calculated in the order of “practical experience” (0.4), “theoretical analysis” (0.3), “knowledge from domestic and foreign counterparts” (0.2), and “intuition” (0.1) (49). The degree of familiarity (Cs) is divided into 5 levels: very familiar (1), more familiar (0.75), average (0.5), less familiar (0.25), and unfamiliar (0). Cr can be calculated by the formula Cr= (Ca + Cs)/2. Generally, a Cr value >0.7 is considered to indicate acceptable reliability.
(3) Coordination coefficient. Kendall's W concordance coefficient test is used to assess the quality of expert consultation and measure the difference in expert opinions on the importance, feasibility and sensitivity of each indicator. That is, the consistency of n experts' scoring results of K indicators at various levels, and the value is 0–1. Statistical significance of Kendall's W test results indicates consensus among experts.

**Table 2:** Judgment basis and familiarity with the topics for consultation from experts.

**Questions**	**Evaluation criteria**	**Your choice**
Judgment basis	a. Practical experience (0.4) b. Theoretical analysis (0.3) c. Knowledge from domestic and foreign counterparts (0.2) d. Intuition (0.1)	
Familiarity	a. Very familiar b. More familiar c. Average d. Less familiar e. Unfamiliar	

In addition to judging Delphi quality from the above aspects, the calculation methods of each index score in this study include (1) arithmetic means of the score for each indicator; (2) consensus rate (or support rate), that is, the ratio with indicator scores 4 or above; and (3) coefficient of variation (CV), which reflects the fluctuation degree of experts' scores on each indicator. The smaller the CV value is, the more concentrated experts' opinions on this indicator are. The accepted competencies were established with mean scores in all three scoring criteria (importance, feasibility, and sensitivity) ≥ 4.0, consensus rate reached 70.0% and a coefficient of variation ≤ 0.25.

## Results

### Basic Information on the Participants

In the first round, a total of 20 consultation questionnaires were issued, and 18 were recovered, with an effective recovery rate of 90%. In the second round, three experts did not give feedback, with an effective recovery rate of 83.33%. Table 3 presents the profile characteristics of the experts. Based on the findings, 18 experts consented to participate in the study from various areas, including Beijing, Shanghai, Jinan, and Hangzhou. Sixty-one percent (*n* = 11) of the participants were from educational institutions, and most had more than 20 years of work experience (*n* = 12, 66.67%). Sixty-one percent of the participants were professors.

**Table 3:** Profile characteristics of the experts (*n* = 18).

**Characteristics**	***N***	**%**
**Age (years)**		
≥30	4	22.22
≥40	5	27.78
≥50	9	50.00
**Years worked**		
≥5	2	
≥10	4	22.22
≥20	5	27.78
≥30	7	38.89
**Professional title**		
Associate professor	7	38.89
Professor	11	61.11
**Workplace**		
Medical colleges	11	61.11
Academic research institutions	4	22.22
Governmental institutions	3	16.67

### Preliminary Results of Delphi Method

According to the calculation, the Cs score is 0.688, and the Ca score is 0.844. Then the value of the expert authority coefficient Cr could be calculated as (0.688+0.844)/2 = 0.767 (>0.7), indicating that the expert consultation results are accurate and reliable.

### Delphi Consulting Results of the Service Capability Evaluation Index System

A total of 35 potential indicators were extracted from the policies and literature, of which 19 were for inputs and 16 were for activities. All these indicators were entered into a Delphi questionnaire to be discussed. In Round 1, 11 indicators did not reach consensus as being “important” or “essential” for inclusion in the core capability framework and were excluded from Round 2. For the indicators recommended by 2 or more experts, they were added in the second round of the questionnaire. If only one expert proposed to add a certain indicator, our research team discussed the indicator and then decided whether to include it in questionnaire 2.0. Based on this principle, an additional 11 indicators were added to the Round 2 questionnaire. In addition, the feedback included the amended wording of four indicators and 2 dimensions. We merged two specific capabilities into 1 under subdimension 2.2 to reduce redundancy (The two indicators of physical examination and physical assessment are combined into one indicator, namely indicator 2.2.1). According to the predefined inclusion criteria, 34 indicators met the criteria and finally entered the second round.

In Round 2, all the indicators that met the predefined criteria remained, and three indicators were eliminated because they were deemed not important and necessary for inputs. After adjusting the indicators according to the first round of opinions, the expert coordination coefficients in the second round were statistically significant in all dimensions (*P* < 0.05), indicating that the opinions of all the experts tend to be consistent (Table 4). Compared with the results of the first round, the average importance score of the secondary indicators increased, and the support rate reached 100%. At the same time, the sensitivity scores of these dimensions were improved, and the coefficient of variation was between 0.000 and 0.147, lower than the results of the first round. The scores of importance and availability of the tertiary indicators were all more than 4, and the support rate increased to 73.33–100%. After completing all the procedures of the Delphi approach, the final core capability framework comprised 31 specific indicators mapped to six subdimensions (i) staffing; (ii) facilities and equipment; (iii) funding; (iv) medical inspection services; (v) health management services; (vi) institutional standard management. The first three subdimensions are the key inputs mentioned in the framework. The fourth to sixth subdimensions reflect both the professional services and internal management of LTCTs, which together constitute the activities in the framework. All the selected indicators are shown in Table 5.

**Table 4:** Kendall's W for indicators in the Delphi process.

**Scoring criteria**	**Round 1**				**Round 2**			
	**W**	**χ^2^**	**df**	***P***	**W**	**χ^2^**	**df**	***P***
**Importance**								
Secondary indicator	0.069	7.398	6	0.286	0.159	11.923	5	0.036
Tertiary indicator	0.204	124.962	34	0.000	0.140	60.002	33	0.003
**Feasibility**								
Secondary indicator	0.393	42.420	6	0.000	0.348	26.113	5	0.000
Tertiary indicator	0.174	106.776	34	0.000	0.374	160.586	33	0.000
**Sensitivity**								
Secondary indicator	0.124	13.399	6	0.037	0.150	11.269	5	0.046
Tertiary indicator	0.156	95.347	34	0.000	0.149	59.068	33	0.004

**Table 5:** LTCF's service capability evaluation index system.

**Primary indicator**	**Secondary and tertiary indicators**	**Importance**			**Sensitivity**			**Accessibility**		
		**Mean**	**%**	**CV**	**Mean**	**%**	**CV**	**Mean**	**%**	**CV**
**1. Inputs**	**1.1 Human resources (Secondary indicator)**									
	1.1.1 Total number of caregivers	4.600	86.67	0.160	5.000	100.00	0.000	4.429	86.67	0.171
	1.1.2 Total number of doctors	4.467	86.67	0.166	4.933	100.00	0.052	4.243	80.00	0.222
	1.1.3 Total number of nurses	4.667	93.33	0.132	5.000	100.00	0.000	4.643	100.00	0.107
	1.1.4 Total number of other technicians	4.200	86.67	0.241	4.933	100.00	0.052	4.267	80.00	0.187
	1.1.5 Ratio of actual open beds to caregivers	5.000	100.00	0.000	4.933	100.00	0.052	4.773	100.00	0.087
	1.1.6 Ratio of nurses to caregivers	4.600	100.00	0.110	4.933	100.00	0.052	4.267	93.33	0.139
	1.1.7 Percentage of certified caregivers at intermediate level and above	4.913	100.00	0.054	4.920	100.00	0.053	4.367	93.33	0.140
	1.1.8 Proportion of nurses with licensed nurse practitioner or above	4.600	100.00	0.110	4.920	100.00	0.053	4.300	93.33	0.138
	1.1.9 Number of doctors in shortage according to qualification standards	4.429	100.00	0.116	4.629	100.00	0.106	4.364	93.33	0.144
	1.1.10 Number of nurses in shortage according to qualification standards	4.514	100.00	0.112	4.700	100.00	0.098	4.379	100.00	0.111
	1.1.11 The professional title of director in LTCFs	4.233	73.33	0.194	4.800	100.00	0.086	4.107	80.00	0.184
	**1.2 Facilities and equipment (Secondary indicator)**									
	1.2.1 Number of registered beds	4.613	100.00	0.107	5.000	100.00	0.000	4.133	80.00	0.180
	1.2.2 Floor area per bed	4.536	73.33	0.175	4.929	100.00	0.054	4.250	80.00	0.177
	1.2.3 Floor area of rehabilitation room	4.533	86.67	0.164	4.867	100.00	0.072	4.253	100.00	0.167
	**1.3 Capital investment (Secondary indicator)**									
	1.3.1 Total annual capital expenditure	4.533	100.00	0.114	4.700	100.00	0.097	4.400	100.00	0.115
	1.3.2 Proportion of annual training expenditure to total expenditure	4.507	86.67	0.149	4.287	100.00	0.137	4.267	93.33	0.139
**2. Activities**	**2.1 Inspection services provided by medical staff (Secondary indicator)**									
	2.1.1 Doctor rounds per week (times/1 week)	4.633	100.00	0.104	4.633	100.00	0.104	4.267	100.00	0.107
	2.1.2 Daily inspections from caregivers (times/1 day)	4.920	100.00	0.053	4.573	100.00	0.108	4.633	100.00	0.104
	**2.2 Health Management Services (Secondary indicator)**									
	2.2.1 Physical assessment for the elderly (times/1 year)	4.433	100.00	0.112	4.267	100.00	0.107	4.040	80.00	0.150
	2.2.2 Nonpharmacological rehabilitation for dementia (times/1 week)	4.713	100.00	0.096	4.133	93.33	0.125	4.267	100.00	0.107
	2.2.3 Nonpharmacological rehabilitation for disabled elderly (times/1 week)	4.707	100.00	0.096	4.207	93.33	0.133	4.273	100.00	0.106
	2.2.4 Update of health records (times/1 year)	4.533	100.00	0.114	4.267	93.33	0.139	4.187	86.67	0.136
	2.2.5 Health education activities (times/1 year)	4.520	86.67	0.145	4.487	100.00	0.112	4.033	80.00	0.152
	2.2.6 Services from cooperative medical institutions (times/1 year)	4.333	80.00	0.188	4.660	93.33	0.107	4.200	73.33	0.205
	**2.3 Institutional management (Secondary indicator)**									
	2.3.1 Service satisfaction assessments from third parties (times/1 year)	4.147	93.33	0.124	4.187	80.00	0.163	4.013	86.67	0.225
	2.3.2 Quality evaluation from third parties (times/1 year)	4.400	100.00	0.115	4.167	80.00	0.168	4.213	93.33	0.183
	2.3.3 Satisfaction assessments within LTCFs (times/1 year)	4.333	93.33	0.142	4.127	80.00	0.202	4.033	93.33	0.165
	2.3.4 Quality evaluation within LTCFs (times/1 year)	4.500	93.33	0.126	4.227	80.00	0.215	4.173	93.33	0.180
	2.3.5 Whether to set up standardized care guidelines	4.867	100.00	0.072	4.600	100.00	0.110	4.447	86.67	0.165
	2.3.6 Percentage of performance rewards for caregivers	4.387	93.33	0.119	4.380	93.33	0.141	4.240	80.00	0.170
	2.3.7 Whether there is a post emergency physical assessment	4.553	100.00	0.110	4.507	100.00	0.111	4.267	86.67	0.165

*Mean, mean score of each indicator from expert panel; %, consensus rate; CV, coefficient of variation*.

## Discussion

With the progress of aging, the LTCI pilot program marks the beginning of systematic development of the China LTC system, covering 49 pilot cities thus far. As mentioned above, only the seniors with a disability level 4 and above can apply for services from an LTCI-designated institution. How can LTC services be better delivered to improve the health outcomes and physical function of the elderly? It is a major but difficult issue that the current LTC system needs to address. The government departments, research institutions, and many other parties have conducted extensive discussions on this topic. To the best of our knowledge, this is the first study focusing on the development of comprehensive capability indicators for LTCFs in the context of the China LTC system, which should be further tested by similar studies in other countries for its validity.

External regulation and the market environment have an impact on the development of LTCFs, urging these institutions to optimize the management process to provide professional and standardized LTC services, which poses a greater challenge to service capability. Most previous studies developed indicators based on the “input” dimension. In other words, it is considered one-sided and unreliable when equating static resources within the institutions with service capabilities. Alternatively, some studies confuse the evaluation of service results with service capabilities. How to transform the realization of core functions into measurable indicators is a difficult point in research. The service functions and positioning of LTCFs in China are still being explored and discussed, and the evaluation index system should be oriented and operable to identify the general advantages or disadvantages of LTCFs. The framework not only emphasizes the static capabilities presented by resource inputs but also fully embodies the professional service and management process based on the inputs. After two rounds of the Delphi process, we pioneer the new indicator framework of service capability, including 2 dimensions: inputs (staffing, facilities and equipment, funding) and activities (medical inspection services, health management services, institutional standard management). The “activities” is built based on the consideration that the service process and internal management play important roles in service improvement. In general, LTCFs tend to be more compliable to the better use of resources when they have a deep understanding of dynamic capabilities and competitiveness, thus making the process of services more appropriate. Therefore, the evaluation of service capabilities should not only concern the static resource input. In the early days of the development and application of dynamic capability theory, some experts in the medical field put forward corresponding views (50, 51), but there is a lack of corresponding discussions in the field of LTC.

The indicators established in this research are further discussed from two aspects. On one hand, with the implementation of the domestic pilot of LTCI, the management departments have developed basic qualification requirements for the resource input of designated LTCFs. In addition to the regulations on the number of personnel, it also considers the configuration of the personnel structure. Regarding the dimension of inputs, we consider that the absolute and relative amounts of human, material and financial resources are common in the provision of services, of which the qualifications of professionals would have a positive effect on the quality of care. The selection of existing indicators is effectively connected with national norms.

On the other hand, we hold the point that the “activities” based on the core input resources is an important component of the proposed framework, which aims to cover various aspects of the care process. LTCFs provide services for people with disability and dementia who have a higher degree of LTC dependency. The professional services in these LTCFs are different from traditional life care services and are of great value for maintaining the physical function and health status of the elderly. The selected indicators, such as non-pharmacological rehabilitation services for elderly individuals with disability and dementia, are beneficial for reducing the occurrence of adverse events (such as falls and infections). The indicators are consistent with the focus of the current LTC disability grade assessment and are applicable to the whole country. On the part of institutional standard management, the internal satisfaction evaluation, quality inspection, and other indicators consistent with the national policy orientation are considered. The service frequency reflects the degree of importance these institutions attach to the improvement of service capability. At the same time, it covers the consideration of other standardized management measures such as regulations and incentives. In summary, based on the theoretical framework and the macro external environment (such as policy norms, institutional requirements, and market demand), the scope of service capabilities mentioned in this study covers the “inputs” and “activities” of the LTCFs. They are all key factors in the formation of service outcomes. Similar to the index system constructed in this study, the Organizational Capacity Assessment Tool User Guide (V.2.0 2016, funded by the European Union) and the organizational capability of hospitals developed by Jingyu Shi both adopted the idea of comprehensive rather than single-dimensional evaluation (52).

In general, the evaluation index system conducted in this study, combined with the background of the LTCI pilot program in China, considers the specific aspects of LTCFs and more comprehensively reflects their service capability. In the initial stage of LTC practice in China, it is feasible to collect data from LTCFs based on the indicators involved in this study. These indicators are usable for identifying the aspects that need greater focus and giving priority to certain improvements. This is a reliable reference for LTCF management, who have a big-picture understanding of overall operations to ensure the continuous improvement of service capability. International LTC practices attaches great importance to the concept of “person-centered care.” Developed countries such as the United Kingdom, Sweden and Australia have gradually developed and applied the observable evaluation indicators, which reflect various subjective dimensions that are difficult to measure (53–55). With the improvement of LTC practice in China, further attention should be paid to the collection of relevant information from the user's perspective, such as the satisfaction of the elderly and their families, the service process that respect recipients' privacy and their living atmosphere. These contents are closely related to service capabilities and should be achieved through service regulation and professional training.

In the next step of the study, we will make a questionnaire to collect data from electronic records based on the final set of indicators and compute scores of service capability in LTCFs with appropriate statistical methods (comprehensive evaluation methods such as fuzzy Borda method). This is the conversion of scattered indicators into quantitative scores that can be compared between institutions. Combining empirical data with specific evaluation methods can not only obtain the total score of LTCF's service capability, but also obtain the score of each indicator in different dimensions. Feedback is sent back to the managers of the sample LTCFs and the relevant administrative authorities. This is a prominent manifestation of the practical value of this study, that is, it helps practitioners to identify the strengths and weaknesses of service capabilities. Therefore, service improvement plans are formed based on gaps in key dimensions. At the policy level, targeted support programs can be explored for the indicators that are generally weak in most LTCFs. We believe that aiming at the improvement of service capability will lead to improved service outcomes. An unavoidable limitation of this study is that some of the indicators should be moderately up to date to reflect the ever-changing development progress in the Chinese LTC system. In addition, the relatively small number of sample experts included in this study, although it meets the basic requirements of the Delphi method, is also a limitation that should be mentioned. If service providers or other stakeholders (experts by experience) are included in the panel, their views may add valuable information to our evaluation tool. In the future, by combing the front-end evaluation of service capabilities with the quality evaluation that emphasizes service results, more targeted and comprehensive institutional management strategies can be formed, which will help promote the progress of the LTC system.

## Conclusion

In this study, the Delphi method was used to construct an evaluation index system for service capability that would be suitable for LTCFs. This is a relatively new perspective for developing indicators according to the characteristics of LTCFs in China. The set of indicators is supposed to quantify and visualize the gap between better LTC practice and policy guidance. They can also provide us with a comprehensive understanding of the current situation of LTCFs' capabilities in China, thus proposing a clear direction for improvement. The combination of indicators with empirical survey data is anticipated to be helpful to managers of LTCFs, government administrators, researchers, and others who want to make decisions, policies, and changes based on the information. Follow-up research can further explore the relationship between service capability and service outcome improvement.

## Data Availability Statement

The raw data supporting the conclusions of this article will be made available by the authors, without undue reservation.

## Author Contributions

WL: conceptualization, methodology, software, data curation, investigation, visualization, and writing—original draft. MH: conceptualization, supervision, and writing—review and editing. WC: supervision and writing—review and editing. All authors read and approved the final manuscript before submission.

## Funding

The study was funded by the Ministry of Education in China Project of Humanities and Social Sciences (Project No. 18YJC630048) and Taikang Yicai Special Fund for Public Health and Epidemic Prevention and Control.

## Conflict of Interest

The authors declare that the research was conducted in the absence of any commercial or financial relationships that could be construed as a potential conflict of interest.

## Publisher's Note

All claims expressed in this article are solely those of the authors and do not necessarily represent those of their affiliated organizations, or those of the publisher, the editors and the reviewers. Any product that may be evaluated in this article, or claim that may be made by its manufacturer, is not guaranteed or endorsed by the publisher.

## Footnotes

^1^Examining Competencies for the Long-Term Care Workforce: A Status Report and Next Steps.

^2^Examining Competencies for the Long-Term Care Workforce: A Status Report and Next Steps.
